# On Experiential Loneliness

**DOI:** 10.1007/s11245-023-09936-z

**Published:** 2023-06-13

**Authors:** Philipp Schmidt

**Affiliations:** Institut für Philosophie, Julius-Maximilians-Universität Würzburg, Ehrenhof Südflügel, Residenzplatz 2, 97070 Würzburg, Germany

**Keywords:** Loneliness, Pluralism, Phenomenology, Connection, Borderline personality disorder

## Abstract

Presumably, everyone has, at some point in their lives, felt lonely. Loneliness is, in that particular sense, omnipresent. What it feels like to be lonely can, however, vary significantly. Loneliness is far from being a homogeneous phenomenon. Different kinds of loneliness need to be distinguished, considering its causes, contexts, a person’s capacities to cope with it, and many other factors. This paper introduces the notion of a specific kind of loneliness: *experiential loneliness*. Experiential loneliness, it will be argued, consists in particular ways of experiencing the world, oneself, and others. Although feelings of being lonely in one way or another can emanate from one’s experience of the world being structured in a particular manner, such kinds of loneliness need not—at least, not always and the whole time—lead to emotional feelings that are concerned with one’s loneliness or the lack of meaningful social relationship. Loneliness can give rise to quite different emotional feelings that sometimes even cover up their provenience from underlying experiential loneliness. The notion of experiential loneliness, it is suggested, helps to tie back certain styles of thinking, desires, feelings, and behaviors to contexts of loneliness. Moreover, it will be argued that the notion can also elucidate the development of feelings of being lonely in contexts in which others are not only around but also available. To develop and enrich the notion of experiential loneliness as well as to exemplify its usefulness, a closer look will be taken at the case of borderline personality disorder, a condition in which sufferers are often plagued by loneliness.

## Introduction

Loneliness is a complex emotional experience that, in one way or another, everyone has felt at some point in their lives. For many, loneliness arises for discrete periods, during specific phases in their lives, or in particular settings or locations. For some, loneliness is a more pervasive condition, accompanying their lives for many years or even decades. Typically, loneliness is associated with emotional distress and often even long-lasting psychological pain, or what some have called “social pain” (Cacioppo and Patrick 2008, p. 7). The COVID-19 pandemic has provided an uninvited and vivid example of how loneliness can affect our experiential lives (Dahlberg 2021). The health implications of chronic loneliness have been well-documented (Hawkley and Cacioppo 2010), highlighting the need for further investigation of this pervasive issue worldwide (Surkalim et al. 2022). Numerous studies have identified various existential, affective, personal, cognitive, biological, developmental, social, cultural, and societal factors that can increase the risk for or cause loneliness (McHugh Power et al. 2018). The causal antecedents of loneliness are as diverse as the contexts in which people can feel lonely. Life events such as losing a close other through ending a relationship, moving to another place, or death can trigger loneliness. Loneliness can also develop in otherwise stable relationships where partners have different working hours that undermine meaningful exchange, or when significant others are emotionally unavailable. Social isolation can trigger loneliness, and the contexts can vary widely, including the elderly living alone at home with few contacts (Smith and Victor 2019), illness (Yanguas et al. 2018), migration (Jang and Tang 2022), and certain professions that are associated with a relatively isolated lifestyle, such as farming (Holton et al. 2022). These few examples suffice to illustrate the heterogeneity of contexts in which loneliness may become an issue.

Can we grasp and explain these various phenomena of loneliness through a single concept of loneliness? Due to the ubiquitous nature of loneliness, the multitude of studies and disciplines involved, the numerous causes of loneliness, and the wide range of contexts in which loneliness can occur, it should not be unexpected that conceptualizations of loneliness are manifold and diverse. Given the multitude of proposals regarding loneliness and its many facets, some have stressed the importance of establishing a standardized definition and operationalization of loneliness, as this will facilitate detecting and monitoring the prevalence of loneliness within a specific population (Surkalim et al. 2022). While the intentions of standardizing the definition of loneliness are understandable and may serve limited purposes, they are unlikely to provide a detailed and profound understanding of the various conditions of loneliness.

Some scholars have attempted to synthesize the different analyses of loneliness to reach a more comprehensive view and have highlighted the need for an integrative concept (McHugh Power et al. 2018). “Conceptual clarity”, they argue, is expected to be most likely attainable when one examines “what individuals *experience as loneliness*” instead of grounding our understanding of loneliness “on prototypical or stereotypical understandings of the concept” (McHugh Power et al. 2018, p. 229). Suggesting an inquiry into the experiences relating to loneliness appears to be a sensible approach: Given that loneliness typically, at least at some point, involves *feelings* of being lonely in one way or another, examining the structure of these feelings will certainly be informative. Moreover, it is precisely our concrete experience of the world in which the different causal factors and contexts can interact and merge *qua* phenomenological aspects and integrate into one unified style of experience (Schmidt 2018, 2021a). Still, do McHugh Power and her colleagues (2018, p. 229) have it right when they conclude “that a philosophical phenomenology approach will help clarify the path” to conceptual clarity?

A few caveats remain, or so I shall argue. For one, we should be careful not to raise false hopes. Aiming for conceptual clarity through a discussion of individual experiences of loneliness is one thing; finding a concept that covers all the loneliness phenomena is another. Consider the following statement by a recent approach to loneliness from a phenomenological perspective: “There are innumerable variables to situatedness that can come into play and contribute to the duration and intensity of loneliness […]. And these variables, taken together, can determine how acute and wide-ranging the experience can be.” (Aho 2022, p. 3) One consequence of the myriad ways in which our lifeworld is constructed is that loneliness can manifest in such heterogeneous manners that a single all-encompassing concept of loneliness may not be possible. Now, there is no need to rule out the possibility of an all-encompassing definition, and perhaps there is a way to construe a taxonomy of loneliness, as some have tried (Weiss 1973). Yet, simply presupposing the possibility of a unified understanding of loneliness carries the risk of overlooking the subtle differences between distinct types of loneliness.

Taking the phenomenological analysis of ‘what individuals experience *as* loneliness’ as a way towards conceptual clarity about loneliness, as McHugh Power and her colleagues suggest, can falsely translate into the assumption that loneliness only occurs when a person’s situation is experienced *as* loneliness. Loneliness is likely to be primarily experienced in a pre-reflective way of emotionally “feeling towards” (Goldie 2002) the world. However, experiencing one's situation *as* loneliness still requires a reflective act and judgment. Surely, examining what individuals experience *as* loneliness will evidently tell us something about what is essential to loneliness. But such an endeavor shouldn’t distract us from the possibility that a person may experience the world in such a way that is best described as an experience of loneliness without the person actually being aware of their loneliness. It seems plausible to assume that one can suffer from loneliness without experiencing one’s situation *as* loneliness. Sometimes, it may only be retroactively or over time that one comes to see that one has been feeling lonely. Some people may have lived lonely lives without ever realizing their loneliness and its impact on their situations. Our experiences *of* loneliness, thus, likely transcend what we experience *as* loneliness.

Developing this thought further, one might ask whether experiences of loneliness are also structurally homogenous. According to what has been labelled the standard account, loneliness is an *intentional* experience concerned with the lack of relevant social connection (Seemann 2022). While this may be true in many cases, it is unclear whether loneliness must always manifest in experience as an intentional state directed at a loneliness-related object or theme.

It is important not to rule out that loneliness may manifest in experience in more vague ways without necessarily developing into an explicit and intentional experience of loneliness. In recent decades, it has been pointed out that moods or “existential feelings” (Ratcliffe 2005, 2008) make up a great deal of our experience of the world but are not intentional in the traditional sense. Unlike intentional emotions, these affective experiences are considered non-intentional in that they are not directed at specific objects or events of the world but are concerned with the world and a person’s situatedness in it as a whole. Related feelings can be fuzzy and have no clear themes. It may well be that loneliness can affect our experience of the world in these more subtle ways.

Consequently, when undertaking the task of compiling a phenomenology of loneliness, the inquiry should not be limited to what we experience as loneliness or to intentional experiences in which loneliness is the object. This is not to suggest that an entirely new concept of loneliness is required. However, existing accounts should be expanded by analyzing those affective experiences that are fuzzy, shape the background of our experience of the world, and are loneliness-related in that they can potentially motivate explicit feelings of loneliness. I propose, therefore, that we should not only study how it feels to be lonely when people experience themselves as lonely but also examine the experiential conditions that can give rise to such feelings of loneliness. This paper aims to achieve this goal.

I have adopted a pluralist understanding of loneliness in writing this paper, recognizing that it can manifest in various structurally heterogeneous ways in experience. Drawing from existing accounts of loneliness, the paper introduces the notion of a specific kind of loneliness. This kind of loneliness, it will be argued, consists in certain styles of experiencing the world, oneself, and others. These experiences may or may not be accompanied by feelings of loneliness. For instance, a depression-related flat affect may impede any deep or meaningful feeling, undermining the general ability to feel a strong connection with others. I refer to this type of loneliness as *experiential loneliness* because it arises from an individual's unique experiential structure and their general way of relating to the world and others. In such cases, feelings of loneliness may emerge as a result, but are not necessarily always present. The notion of experiential loneliness, with its focus on a person's general style of experiencing the world and relating to others, can shed light on cases where individuals experience loneliness even when others are available in a relevant way. In other words, the concept of experiential loneliness is not only applicable to situations where a lack of social connections is apparent, but also to cases where the individual's experiential structure gives rise to feelings of loneliness despite the presence or availability of others. First, Sect. 2 spells out the pluralist view of loneliness that forms the background for developing the notion of experiential loneliness, which will be the theme for Sect. 3. Section 4 further discusses the notion in light of borderline personality disorder to illustrate how the concept works and how it can help understand the relationship between experiential styles and the genesis of feelings of loneliness.

## What is Loneliness? A Pluralist View

In her book *Biography of Loneliness*, Alberti (2019, p. 21) emphasizes that the concept of "loneliness" is a modern invention, which has replaced the more traditional notion of "solitude" that simply referred to being physically alone. Solitude was often associated with positive features, as it would allow a deeper connection to God or oneself and ultimately even “enable the individual to fare better in society” (Alberti 2019, p. 22). Modern loneliness, by contrast, is associated with emotional distress. As Arendt described with her famous acumen: “In solitude […] I am ‘by myself’, together with myself, and therefore two-in-one, whereas in loneliness I am actually one, deserted by all others.” (Arendt 1962, p. 476) Solitude and loneliness do not simply differ in that one, solitude, describes objective circumstances, whereas the other, loneliness, refers to subjective circumstances, i.e., specific subjective experiences, as it has sometimes been implied (Ma et al. 2020). Both labels most certainly cover two distinct sets of experiential phenomena that need to be distinguished phenomenologically. My concern in this paper is only with loneliness.

### The Standard View of Loneliness

Can we identify a central phenomenological structure pertaining to all the various experiences that fall under the label of experiential loneliness? Take, for instance, what has sometimes been described as the standard account of loneliness, a widespread view according to which loneliness has three features: (1) it is an experience; (2) it is associated with unpleasant or painful, in any case, negatively valenced feeling; (3) it is an intentional experience in that it is about or directed at the discrepancy *between* the quality or quantity of one’s factual social relationships *and* those that would be required to fulfill one’s social needs (Seemann 2022). Even if we were to accept such a formal and all-encompassing concept of loneliness, would this suffice to pin down the phenomenological essentials of experiences of loneliness?

I argue that we should be hesitant about answering the question in the affirmative too quickly. On the one hand, the concept is indeed vague enough to cover many cases. But on the other hand, this vagueness comes at the price of superficiality, resulting in that it doesn’t really help to understand what it is like for an individual to feel or be lonely. Defining an object by saying it is spatiotemporal and is prone to be found in waters like seas, lakes, or rivers, doesn’t help us discern which object we are talking about. Such a comparison is evidently exaggerated, but I fear something similar might apply to the standard view of loneliness. Try to empathize with a person who feels lonely after a painful breakup with their partner and the feelings of being lonely that can arise in the aftermath. Imagine that you didn’t initiate the breakup and that you feel lonely in terms of being left alone. Or, consider that you have ended the relationship because you no longer feel any connection with the person you had been living together with for so long. Are the pain experiences of loneliness in both cases approximately the same?

Consider, for instance, the loss of a partner in a tragic accident. This may be someone with whom you have shared a healthy, functioning relationship, and whom you consider your true “soul mate”. The disruption to your life is profound, as you feel that the most meaningful connection you have ever had with another person has been torn away, leaving a void that you feel cannot be filled by anyone else, now or ever. The resulting feelings of loneliness are likely to be intense. Can these feelings be compared to the loneliness experienced by someone who endures a painful indifference to their partner?

Similarly, imagine a child who must stay home due to illness while their friends are away at summer camp. How does the loneliness they experience compare to that of an older adult who, despite having enjoyed many meaningful and fulfilling relationships, is now isolated due to various age-related factors and is aware that they will never again be able to form new connections? It seems to me that these different kinds of loneliness cannot simply be lumped together under a single label.

### Enriching the Standard View: Pro-Attitudes in Loneliness

But what if we enriched the standard view by adding a more detailed characterization of the kind of intentional attitude found in loneliness? Consider Roberts and Krueger’s (2021) account, which describes the intentionality of loneliness in terms of its two general components: the specific “formal object” (Kenny 2003, p. 132) of the emotion of loneliness and a specification of the attitude involved. According to the model, not far from the standard view, the formal object of loneliness is a perceived lack of the availability of *social goods* “such as companionship, moral support, physical contact and affection, sympathy, trust, romance, friendship, and the opportunity to act and interact—and so to flourish—as a social agent” (Roberts and Krueger 2021, p. 7). Moreover, the model emphasizes that loneliness involves a *pro-attitude* toward others and the specific social goods they can provide. In other words, loneliness encompasses more than just negative feelings; it also includes a yearning for social interaction and a desire to connect with others. That evidently further adds to the standard view as described, for do not all the provided examples involve a felt desire to be with others, a longing of sorts? Certainly.

Even though this very plausible and helpful account of loneliness can illuminate much about what loneliness often means for the sufferers, I still believe that it doesn’t cover all the phenomena that should run under the label of loneliness. To explain, firstly, adding the desire component only, i.e., the pro-attitude, will still not suffice to mitigate the worries that one oversees the heterogeneities found in different emotional feelings of loneliness, as longing can—similar to pain experiences—vary significantly. Secondly, the “frustrated pro-attitude account”, as Roberts and Krueger (2021, p. 15) also call it, defines loneliness through its formal object, the unavailability of social goods. According to Roberts and Krueger, the frustration of a pro-attitude towards certain social goods triggers an emotional experience of loneliness directed at the unavailability of social goods.

However, one might ask whether loneliness always has an explicit object of any kind to begin with. Consider, for instance, you move to a new city or country. It’s tough. You don’t know anybody. One Friday night, you open a bottle of wine at home alone, and you realize you are alone. You think about past times with your friends, how you miss them, and how you miss having friends. You are aware of the lack of connection and feel sad about it, experiencing a frustration of the wish to be with others. This clearly is an emotional experience. But now consider, in the weeks after the emotional outburst, you have forgotten about the evening. After all, you are new in town, and things might change over time. Still, you remain in a melancholic mood, characterized by nostalgic episodes of memory and a generally open mind for the future, including for new encounters that must occur if one just continues to make the new life happen. While in this mood, you can enjoy TV shows, get angry over what you read in the newspapers, or feel satisfied with your work progress. In this case, loneliness rather manifests as a mood, in which the lack of available social goods need not be a thematic moment while living through the mood. In fact, one can be concerned with other objects and become emotional in all kinds of ways. The lack of social bonds is not explicit but implicit as the experience of one’s lifeworld unfolds. For instance, one might be more vulnerable and sensitive about how encounters with strangers run. More meaning might be attributed to how people at work respond to oneself, as needs for social affiliation are projected onto the work sphere. A general felt insecurity toward other people and the future might emerge, an insecurity that oscillates between general anxiety, hope, and trust in the future. Now, consider a young child with no friends around for extended periods. Likely, moods and emotions of loneliness are so pervasive that they become hard-wired into the child’s affective architecture to the effect that its whole world seems to essentially lack the possibility of enjoying the social goods it needs. This may result in that expectations towards others sediment into a specific, more pervasive “existential feeling” (Ratcliffe 2005, 2008) which is characterized by a sense of the possible in which meaningful encounters and connection with others are not included, rendering the world as a whole for the child a lonely place.

Now, my point is not that loneliness qua *emotion*, qua *mood*, or qua *existential feeling* are three completely independent phenomena. Indeed, they all have something to do with the formal object of loneliness-qua-emotion: the unattainability of relevant social goods. However, not all related experiences have the formal object of loneliness as their explicit theme. Especially in the case of existential feeling, loneliness is a rather diffuse affective experience that structurally underlies any more pronounced intentional directedness at some object (Ratcliffe 2005, 2008). Loneliness need not always manifest as a discrete emotion intentionally directed at the lack of bonding with others. Hence, even the enriched standard view should be extended to better account for the full scope of loneliness experiences. I will now briefly sketch such a reconfiguration, a pluralist view that will also allow me to address what I will introduce as *experiential loneliness*.

### Turning the Enriched Standard View into a Pluralist View

A pluralist view on loneliness, as I envision it, has one great advantage: it alleviates the burden of trying to encompass all possible phenomena of loneliness within a single description, allowing for a more nuanced understanding of the various manifestations of loneliness. What are the main assumptions of a pluralist view? Let me sum up the results of my discussion so far.

#### Causes and Contexts are Part of the Experience

The first assumption is that causes and contexts of loneliness shape the experience of loneliness. The idea is that loneliness as an experience is not reducible to a specific feeling triggered by certain causes and contexts. Instead, depending on the causes and contexts, loneliness can feel quite different, as causes and contexts and the awareness of them inscribe themselves into the experience of a situation.

#### Phenomenological Variety

The second assumption is that different phenomena of loneliness experiences need not share a common phenomenology: i.e., they can be different experiential types, such as emotions, moods, or existential feelings; they need not all have the same phenomenological structure and need not be intentional, to begin with; thus, they need not be object-directed and so are not all directed at the formal object of loneliness-qua-emotion; even if they share the same phenomenological structure, they need not be phenomenologically congruent, as not all loneliness-emotions feel the same, nor do the loneliness-moods and involved existential feelings.

#### Family Resemblance

The third assumption is that phenomena of loneliness form a family insofar as they, in one way or another, *relate* to the formal object of loneliness qua emotion: the lack of social bonds and goods.

How does *experiential loneliness* relate to the formal object of loneliness-qua-emotion? I shall argue that experiential loneliness occurs when a person lacks social bonds and goods because of how they experience the world, themselves, and others. Social goods might even be available, but because of the person’s experiential styles they cannot consume and enjoy these goods. I will now unpack this idea by developing the notion of experiential loneliness further in the next section.

## Experiential Loneliness

To better specify what is meant by experiential loneliness, this section develops the notion by examining various theories of loneliness proposed in the literature. Before doing so, I provide some remarks concerning my terminological choice and the concept of experiential styles.

### Terminological Remarks

In the last section, I have argued for a pluralist view of loneliness, according to which we should distinguish different loneliness concepts that refer to distinct though related phenomena. By introducing the notion of experiential loneliness, I aim to grasp a specific group of loneliness phenomena. What is specific to these phenomena is that feelings of loneliness do not emerge out of factual social isolation but are the result of how a person’s experience of the world is structured. This can be illustrated by comparing a few different cases:

#### Case 1

A is physically alone at their apartment and has no one to call or to meet, as it is too late in the night; they feel lonely in being physically alone and lacking any possibility to change the situation.

#### Case 2

B sits alone in a bar. People around in the bar seem nice and in a good mood. B is interested in them but still feels shy about making the first move. B feels lonely, but perhaps this will change once they have made contact with other people.

#### Case 3

C sits alone in a bar. People around in the bar seem nice and in a good mood. Unfortunately, C is not interested in them, as they don’t feel a sense of belonging to the kind of people around. C feels lonely, but—no offense intended—they don’t even want to connect with the others from the bar.

#### Case 4

D is in a bar with their colleagues from the sports club. They are all nice and friendly, yet D is very nervous and struggles with making conversation with others. For D, it is essential how others think of them. D’s insecurity can only be mitigated if the people around them focus their attention on what D says and confirm them through body language. Otherwise, D suffers from high arousal, sweating, and hard-to-regulate anxiety. Because of this, D feels uncomfortable at the bar, perceives others as a threat, doesn’t talk much, and only says things that will likely be met with affirmation. Although other people D likes are around, D feels cut off from the others, detached, and unable to create or feel a connection.

In cases 1–3, the persons feel lonely primarily due to external reasons. A would not feel lonely if they were able to call or meet their friends; it's just that their friends are currently unavailable. B hasn’t made contact with the people around them yet, but the possibility of connecting with others remains and is experienced as such. C feels general connections are possible for them in the bar, but given the people who are around them, C is pessimistic. Perhaps on another day at another location, things might look differently. D, by contrast, has nice people around them, so connection with the right kind of people is generally available. However, given how D experiences themself, others, and the world, it is hard for D to enjoy the social goods presented to them. While D might experience connection as objectively possible, they might experience it subjectively impossible for themself. I want to suggest that D is an example of *experiential loneliness*. It is D’s experiential style of perceiving the situation that undermines connection with other people. By ‘experiential style,’ I refer to habits, reflexes and dispositions in responding psychologically to specific events and states of affairs. D’s requiring for their own psychological comfort that others confirm what D says in order not to feel undermined in any way is a style of relating to others. And so are extensive needs for approval from others. Feeling vulnerable because one struggles with regulating one’s arousal and related nervous reactions is a style of experiencing oneself in the world.

The term ‘experiential loneliness’ is meant to cover the experiential styles that may isolate a person from others and hinder a person from feeling connections with other people. It should be noted that the term is not meant to distinguish between experiential and allegedly non-experiential loneliness. Indeed, it strikes me as hard to grasp what a state of loneliness that has no manifestation or effect on experience whatsoever could be. Instead, the term is meant to distinguish between types of loneliness that are shaped by how the social world really is on the one hand (cases 1–3) and those types of loneliness that are more determined by how a person experiences the social world on the other (case 4).

### State, Trait, and Existential Loneliness

Before giving more real-life examples in Sect. 4, the remainder of this section is devoted to further developing the notion of experiential loneliness in light of existing concepts of loneliness. First, I shall discuss a threefold distinction suggested by Mayers and Svartberg (2001), who emphasize that theories of loneliness can be divided into three categories: those which define loneliness as (1) a *state*, (2) a *trait*, or (3) an essentially *existential condition*. Instead of reading these three categories as competing groups of theories, bearing in mind a pluralist view, I treat them as different types of loneliness.

State loneliness, on that view, is a transient experience of loneliness which can, on my interpretation, involve emotional experiences, loneliness-related moods, or existential feelings.

Trait loneliness, in contrast, is rather a kind of loneliness that manifests as a characteristic feature of a person. This may be due to the personal biography and/or rooted in specific personal features, e.g., low self-esteem, that are prone to trigger feelings of loneliness. This kind of loneliness, given that it is personality-related, persists over longer time. It is in principle changeable, at least, as far as the relevant personality traits can be altered and substituted by others.

Existential loneliness, on the other hand, is typically understood as the general condition of human existence. It is an existential fact that each individual is alone in a fundamental sense. Everyone enjoys a single experiential perspective on the world, which no matter how good the connection with others is, will never fully merge with the other: the state of spherical people in Plato’s myth described in *Symposium* is unattainable. This existential loneliness is more or less explicitly known by everybody. It is inevitable, and, consequently, cannot be cured. All that remains possible is to try to “escape the sense of being condemned to this frightening state” and to “distract oneself with love relationships” (Mayers and Svartberg 2001, p. 542) or to relate with others who help “us to forget that we are alone” (Tillich 1980, p. 549) in other ways. Existential loneliness thus always lingers at the ground of our experience of the world, causing related feelings of loneliness to be foregrounded whenever the effects of distractions fade out. Such ascending might occur, for instance, when we lose significant others through death or break-ups.

How does experiential loneliness relate to these three types of loneliness? Experiential loneliness refers to styles of experiencing the world, oneself, and others that impede the experience of connection with others. As a result, due to related isolation, social needs remain unfulfilled. Accordingly, experiential loneliness can lead to and set the ground for different forms of state loneliness (later, I will give more detailed examples). However, can experiential loneliness be described as a state? Consider, for instance, that you drank too much coffee and now you feel dizzy, your heart is beating like a drum, your thoughts are rushing, and you are talking too much and too fast in a conversation while barely noticing that the other person would like to say something as well. Such a state might impede real connection with the other, and when the other leaves, one might regret having missed the opportunity for a deeply felt encounter. However, in my view, this state is not yet experiential “loneliness.” Not being able to connect in the most profound way with others happens quite often in everyday life and within the most deeply felt relationships. Sometimes you are emotionally unavailable to your partner because you are focusing on your job, some task or because you have terrible back pain. Some experiences in some contexts can prevent emotional connection. But these are only passing moments, and they concern the quality and content of the experience. What I have in mind with experiential loneliness is more structural and pervasive, as it concerns the general styles of a person in experiencing the world.

How abolut trait loneliness? Since patterns in experiencing the world are more pervasive, I would agree to have some personal styles in experiencing the world identified as personal traits. However, I would emphasize that talk of traits might carry the risk of attributing a general and unchangeable trait of loneliness to a person. So even if certain experiential styles amount to experiential loneliness and constitute personal features, they always run short in defining who a person is and are generally changeable.

The fact that experiential styles can generally change renders experiential loneliness potentially curable, which sets it apart from the notion of existential loneliness. Existential loneliness can, at best, be forgotten by being with others, and so can even be said to motivate bonding attitudes and behaviors. Experiential loneliness, by contrast, hinders any real connection in the relevant sense.

### Individual and Sociological Perspectives

So far, I have thematized loneliness from an individual perspective. In fact, most of the research on loneliness moves within an individualist framework, shedding light on factors triggering loneliness that lie in the individual’s condition or the interaction between individuals. Some, however, have emphasized that how society is structured can play an important role in developing loneliness. Weiss (1973) has been an influential voice in that regard, distinguishing between *social* and *emotional* loneliness. In social loneliness, a person doesn’t have a sense of belonging to a group or community. Emotional loneliness refers to the lack of intimate friends and profound relationships. As Donbavand (2021) observes, several accounts have offered similar typologies and emphasized social aspects of loneliness (Franklin 2009; Hawkley et al. 2008; Locke et al. 2010; Woodhouse et al. 2011). For instance, it has been highlighted that developments since the 1970s in people’s view on marriage and social bonds altered the understanding of the nature of bonds “to the point where norms, expectations and experiences have become very confused” (Franklin 2009, p. 344). Over the decades a transition took place “in which solid relationships characterized by the bond (at work, between partners and so on) have given way to individual freedom and the ‘until further notice’ relationship ….” (Franklin 2009, p. 345) What results from this, in fact, is that relationships, in general, become “liquid” (Franklin 2009, p. 345) to the effect that “there is nothing at all to stop those who love you now, who support you now, who employ you now, from dumping you the minute they become bored of you or find a better alternative” (Franklin 2009, p. 352).

Donbavand, although appreciating analyses of this kind, fears that focus on the quality of relationships may end up resulting in an individualist perspective: “For if relationship quality is of central importance then the capacity of the individual to develop such relationships becomes critical.” (2021, p. 75) His aim is to offer an alternative theory that focuses on a sociological explanation of loneliness by further inquiring into “what exactly it is about certain societies that might contribute to loneliness without falling back on arguments of cultural psychology” (Donbavand 2021, p. 75). His Simmelian-inspired notion of *structural loneliness* ought to provide an answer to the task at hand. The idea is that today’s societies are structured in a particular way that undermines the development of social bonds. What is the structure he has in mind? As a hypothesis to be further empirically investigated, he suggests that “a lack of sufficient opportunity for repeated encounters with the same individuals across differing social circumstances” may “account for reports of unpleasant feelings and dissatisfaction with relationships” and so amount to “a new form of loneliness” (Donbavand 2021, p. 80). Loneliness might emerge when we know too little about the people we are dealing with in our everyday lives, given that we meet others only in terms of social roles: we know the postman only as the postman, the colleague only in their function in the workflow, the cashier as the person to pay for the goods one buys.

How does experiential loneliness relate to Donbavand’s structural loneliness and the distinction between an individualist and sociological framework? First, I want to emphasize that I do not think we need to play out different explanatory perspectives against each other. After all, most likely, both the individual as well as the social sphere will affect how a person experiences their social world. The experience of the social world depends on both the factors that determine an individual's style in processing experiences and the reality of the social world itself. To illustrate, a simple example may help: how a person experiences a bottle on a table depends on the objective features of the bottle and their experiential state. For instance, if the person is very tired, drunk, or drugged, their perception of the same bottle may differ. Now, of course, the social world is not something objectively existent that I simply needed to apperceive as it is. In engaging with the social world I contribute to what social reality looks like and my interaction with others shapes my social relationship. But still, how I can interact with others also depends on how others are available for me, that is, which kind of relationships others are open to and thus what my social environment has to offer. Hence, the experienced quality of relationship is always dependent on all three (1) my perception of the others, (2) their perceptions of me, and (3) the kind of interactions all parties are capable or willing to engage in. The notion of structural loneliness primarily addresses how a society's structure enables or impedes specific interactions (and, by the same token, how people experience each other). The notion of experiential loneliness is primarily addressing how an individual experiences the world, itself, and others (and, by the same token, what kind of interactions with that individual are possible). This includes contexts in which others would be open and available for connection, while the experiential structures of the individual under scrutiny prevent the individual from entering fulfilling relationships.

In this sense, the notion of experiential loneliness has an individualist focus as it aims to explain the phenomena of loneliness by looking into the experiential constitution of the individual. However, it does not deny the necessity for complementary sociological perspectives. After all, the experiential constitution of the individual could be, and often is, rooted in how a society is structured. But the point is, it need not be. Individual styles that prevent meaningful connections can occur in a society that lends itself to forming social bonds. The notion of structural loneliness will fall short of explaining these cases, resulting in the need for complementary notions. ‘Experiential loneliness’ is one such complementary notion. I will now qualify it more positively by discussing how it relates to feelings of loneliness.

### Experiential Loneliness and Loneliness-Related Feelings

When Donbavand (2021) introduces his notion of structural loneliness, he adopts the commonly accepted distinction between the objective state of *social isolation* (a lack of social connections) and *loneliness* as the subjective feelings that can develop due to the absence of meaningful social relationships. Applying this distinction to structural loneliness, he emphasizes:

“Properly speaking, a lack of sufficient opportunity for repeated encounters with the same individuals across differing social circumstances can only be considered a novel form of social isolation, not loneliness. It is only when, or if, this type of social isolation can be shown to account for reports of unpleasant feelings and dissatisfaction with relationships that a new form of loneliness can be said to have been detected. Until then, structural loneliness, as I am provisionally referring to the phenomenon, remains a hypothesis for empirical investigation.” (Donbavand 2021, p. 80).

Donbavand’s point is that there is no problem in assuming that people could nonetheless be satisfied with a kind of life in which relationships are superficial and pre-defined by social roles in very limited ways. That is, even if people might be socially isolated because they know others only in one-dimensional ways, they could still feel sufficiently fulfilled socially. Social isolation alone does not amount to loneliness. Such a kind of social isolation only turns into structural *loneliness* if people develop feelings of loneliness in some manner *based* on that very one-dimensional structure of social interaction.

How about experiential loneliness? Do the styles in experiencing the world, oneself, and others that, according to the view envisioned here, are said to impede social connection with others always amount to experiential loneliness? Or do they rather present a form of social isolation? After all, if a person’s world experience is structured in such a way that meaningful connection is barely or only under particular circumstances possible, then perhaps the person will not crave social connections to begin with, one might assume. However, I think we should be very cautious with any such a priori claims. In fact, I believe it is easy to find cases in which people are isolated due to how they experience the world and crave deep connections with others (I will provide some later). On the other hand, there is no reason to believe that people with such experiential conditions necessarily need to develop cravings for sociality. Individuals vary significantly in their needs to be with others or feel connections with them. Some might develop a felt need for social connection. Others may indeed experience the world around them in a way that renders the development of what is typically considered a meaningful connection with others difficult or impossible, yet they do not feel they are missing out on anything. In such cases, when people do not develop social cravings, although their experiential styles make it difficult to connect with others, I suggest speaking of social isolation rather than experiential loneliness is more appropriate. However, I suspect that cases like these will be rare. Consider autism spectrum disorder (ASD). It has traditionally been assumed that individuals with ASD, especially those with strong interests and hobbies, are not simply cut off from others because of their styles of experiencing the social world but also because they lack the desire for social connection.^1^ Yet, research in the past years has shown that individuals with ASD do, in fact, frequently crave social relationships, even though the desired connections might take forms that are different from relationships between neurotypical individuals (Hymas et al. 2022; Umagami et al. 2022).

To sum up, although I want to retain talk of the possibility that isolating experiential styles need not necessarily lead to feelings of loneliness, I suggest that feelings of loneliness most likely ensue from such experiential styles at some point. Accordingly, I deem two central aspects are characteristic of experiential loneliness:

1. Corresponding experiential styles motivate specific feelings, i.e., they constitute a *tendency* towards the development of feelings of loneliness;
2. They need not always and all the time lead to actual feelings of loneliness, as the tendency towards the development of feelings of loneliness can also trigger quite different kinds of feelings, cognitions, and behaviors that need not have the lack of social connection as an explicit, intentional content nor the qualitative feel of being lonely nor are perceived by others as clear expressions of loneliness.

Thus, on my take, there *is* an intrinsic relationship between experiential loneliness and loneliness-related feelings. But it doesn’t imply perfect correlation or congruence. The inherent relationship consists in the former’s tendency towards the latter, insofar as being impeded by one’s own style of world experience to build fulfilling social relationships will ultimately result in falling short of developing the social bonds needed for personal happiness. Moreover, even within social bonds, it can occur that, precisely because of a personal style in experience, contact with others remains dissatisfying. Consider the frustration of having burned your tongue with hot soup and then missing the taste of the main dish. The dish is there, but you are unable to consume it properly. Even with illustrations like these, however, until this point, my discussion has been primarily conceptual and abstract. In the following section, I will discuss examples of experiential loneliness in more detail.

## Examples of Experiential Loneliness

Experiential loneliness has been introduced as a particular style of experiencing the world, oneself, and others, which undermines the development of fulfilling social relationships or encounters. But what exactly does this style—phenomenologically speaking—actually look like for the person embodying it? The first thing to note is that it is not really just one style, but rather a group of different variants that can have a similar effect: causing one to lose touch with the other. The way in which social deprivation, despite real contact with others, experientially unfolds can vary and take many forms. I will now turn to some real-life examples to illustrate possible phenomenological aspects of experiential loneliness and to enrich the notion.

### Borderline Personality Disorder

Borderline personality disorder (BPD) is undoubtedly among the most painful and impactful of all mental health issues. It also presents a particularly complex clinical picture. Typically, sufferers struggle with three core issues: instabilities in affect, self, and social relationships (cf. Schmidt 2020, 2021a, b; Schmidt and Fuchs 2021). Related phenomena include a lack of self-feeling, identity confusion, inner emptiness, affective impulsivity, emotional dysregulation, and recurring interpersonal problems. Often, individuals with the clinical picture of BPD tend to become embroiled in turmoil with their closest friends and family, frequently experiencing sudden, repeated, and particularly hurtful disruptions in relationships. Many of these disruptions result in the permanent end of relationships, while others require periods of repair. In both cases, sufferers undergo phases of strongly felt loneliness, often also manifesting as feelings of abandonment or craving for social connection and attention. This should come as no surprise since when significant social relationships are under threat or get disrupted, most people would eventually develop feelings of loneliness.

My interest in this paper, however, is not with the feelings of loneliness that might accrue after factual losses but rather with those that can emerge even when people are within a relationship and in the presence of the other. These feelings of loneliness are less explicit and mixed together with different feelings, such as emptiness, loss of control, and a general sense of woundedness. Together, they manifest as an “unbearable mental pain” (Fertuck, Karan and Stanley. 2016, p. 2) or “desperate vitality” (Stanghellini and Rosfort 2013), as some have circumscribed the feeling lingering at the background people with BPD often express. These feelings can emerge even in the presence of important others. While in most people, similar feelings of the pain of being lonely can lead to opening up and, even better, connecting with others (Cacioppo et al. 2014), BPD sufferers precisely struggle with the latter. But why? What are the reasons behind the difficulties that individuals with BPD face in connecting with others or maintaining established connections, despite their desire for it? And, what do the difficulties consist in, in the first place? Obviously, there is no one central and comprehensive answer. In fact, as I want to suggest, there are different aspects characteristic of the styles in experiencing world, self, and others, which BPD sufferers enact, that can undermine felt connection.

#### BPD-Related Affective Styles

Let me start by giving a few examples regarding the style of processing emotional feelings and other affective phenomena. Processing emotional feelings is a central aspect of BPD, and sufferers typically struggle with regulating their emotions. Several related phenomena shape how emotions unfold in borderline experiences. First, people with borderline tend to show *alexithymia*, i.e., they struggle with identifying, labeling, or understanding their own emotions (Loas et al. 2012). Second, they are prone to *emotional contagion*, i.e., they quickly catch up on the emotional feelings of others and cannot distinguish their own from the others’ emotional process (Salgado et al. 2020). Third, they are often tensed by *hypersensitivity*, i.e., they strongly focus on emotional cues in others, trying to become aware of how others feel and to detect potential changes therein (Frick et al. 2012). Fourth, they tend to affective *impulsivity*, i.e., they often experience powerful emotional pulls that are hard to control and have the character of an emotional flooding (Sebastian et al. 2013). Fifth, they perceive the *locus of control as external*, something they are forced to respond emotionally to (Hope et al. 2018).

Now, all these aspects of affective style are well-documented in BPD. Elsewhere, I have argued that they translate into a form of social impairment (Schmidt 2022). Do they also relate to loneliness? This is not, at least not immediately, evident. Yet, this is precisely what I want to suggest. To argue that these aspects undermine connection and so present a form of experiential loneliness, Fuchs’ notion of “interaffectivity” (Fuchs 2013), can be helpful. Typically, when we are emotional and are in the presence of other people, our feelings do not simply run off purely in private. Rather, we show how we feel by the way we talk, gesticulate, or through our facial impressions and bodily postures. And others do too. Thereby, a shared emotional space is constituted in which people’s affective processes play into, communicate, and influence each other. Instead of two or more disparate and independent affective processes, we typically engage emotionally in interaffective spheres.

The notion of interaffectivity can also help to elucidate social connection. Connection arguably is a feeling that involves a sense of being in touch with the other. When we feel connected, we experience certain ways of interaffectivity with others, which involve an emotional exchange that allows us to feel synchronized with the other person in a relevant way. We feel emotionally understood and met in the way we feel. Furthermore, we reciprocate by having a sense of how the other person is feeling and why they feel that way, and we show relevant responses to their feelings. Feeling connected, we need not undergo exactly the same feelings as the other persons. When we share our own feelings and join others in their emotional process, an exchange takes place. This exchange does not dissolve the poles of feeling that each of the involved persons embodies. In fact, it is precisely the exchange between different feeling bodies through which we jointly constitute and shape the interaffective space that we inhabit. Connection can only be built out of the structure of interaffectivity and involves the establishment of specific forms of interaffectivity. For, certainly, not all interaffective spaces foster felt connection. The BPD-related affective styles mentioned, my argument now goes, disturb processes of interaffectivity and so undermine the development of the specific interaffective structures that could possibly amount to felt connection. How do these affective styles shape interaffectivity?

##### Alexithymia

Lacking a sufficient understanding of what exactly and why one feels the way one is feeling makes it hard to feel understood and met by others too. Struggling with finding out more about one’s own emotions, the responses of others cannot be evaluated. In fact, typically, others’ responses are often perceived as irritating and unfitting by BPD sufferers, and they will likely feel misunderstood.

##### Emotional Contagion

The tendency to pick up the emotional feelings of others (or rather, what people take others to feel) similarly hinders real emotional encounters and exchange. For, emotional contagion further undermines affective self-understanding and blurs the I-Thou boundary. BPD sufferers either feel consumed by the others’ affects or feel alienated by unexplainable foreign feelings that cannot be recognized as belonging to the other. No felt connection between different yet related feeling subjects can be established. Instead of a shared interaffective sphere, a diffuse emotional space develops.

##### Hypersensitivity and External Locus of Control

The intense focus on emotional cues in others' behavior and bodily expression, coupled with a tense directedness at them, exacerbates the effect of emotional contagion. Rather than promoting affective self-understanding and communication with others, BPD sufferers enter a mode of detection-disposition, responding to detected shifts in the emotional state of others and managing the situation by handling their feelings. Because they are intentionally focused on the emotions of others, they perceive others' actions as a result of their own actions, often as provocations or tests, rather than as expressions of communicating, autonomous feeling subjects.

##### Impulsivity

Impulsivity has a two-fold intrinsic disruptive impact on BPD sufferers. Firstly, sudden and intense emotional shifts can cut off the person from any shared moment with others, leading to an immediate isolating and distancing effect. The severity of the emotion and its eruptive and flooding character make the person feel alone in this process and separate them from others, who do not experience their feelings at the same level of intensity. Secondly, when there is a significant discrepancy between the level of intensity of one’s own feelings and that of others, the way others respond to the situation is seldom perceived as accurate. This makes it difficult to repair affective and interpersonal synchrony, leading to a feeling of fundamentally disagreeing about most aspects of a shared situation.

BPD sufferers' phenomenological style of experiencing affect during acute phases makes it nearly impossible to engage in emotional exchange that could give rise to feelings of connection. The affective processes they experience are inherently isolating and can lead to profound feelings of loneliness. More examples of aspects of experiential loneliness can be given when we consider styles in self-experience and social intentionality.

#### BPD-Related Styles in Self-Experience

Instabilities in BPD also concern people’s self-experience and sense of identity. To illustrate how aspects of these can undermine felt connection, it will be enough to pick out two prominent aspects of borderline self-experience. First, people often are haunted by an excruciating lack of self-feeling: “I feel, for the most part, that I am only just existing. I am part of a continuum but no more, potentially less”, writes Topher Edwards, a BPD sufferer, in his diary (Edwards 2015, p. 14). Others express similar feelings by saying they are “feeling nonexistent” (Black et al. 2014, p. 80) or “deadened” (Singer 1987, p. 133). Such feelings of self-nihilism clearly do not invite feelings of connection. In fact, they consume any potential felt meaning and render the whole world experience meaningless. Sufferers may still experience that other people are capable of meaningful experiences, but for them, these are deemed impossible. Lack of self-feeling, therefore, also includes the experience of the absence of the other, as losing touch with oneself implies losing touch with others.

A second aspect of borderline self-experience is the tendency to overidentify with specific roles, especially the role one has or deems relevant in a given situation. People typically have different roles in society, as we are fathers, sisters, co-workers, lovers, friends, role models, students, or customers, among many others. Typically, despite many divergent roles, people tend to have a unified sense of identity, one that allows a person to be a caring, sensitive mother, an eloquent and powerful businesswoman, and an empathetic friend. Given a generally unstable self-feeling, BPD sufferers struggle with a unified sense of identity that would be robust enough across different situations and personal roles. In fact, they often struggle with synthesizing various aspects of themselves and so tend towards taking one of their roles as absolute, overemphasizing something as their full identity, which is only one side of them. In doing so, aspects of other sides of their identity are experienced as alien or irritating, and the commitments that adhere to the many roles are rejected, denied, in any case, not felt. This has tremendous repercussions for the experience of connection: the different roles and identities that are felt as alien and not belonging to oneself involve relationships with other people. Once the roles and identities are alienated, so are the corresponding relationships that are no longer perceived as concerning oneself. BPD sufferers, therefore, tend to develop a mask in their interaction with others: “To be honest, I am growing tired of this masquerade. … I go through life doing what needs to be done to fulfill my role in society”, says Edwards (2015, p. 49). Developing what has been called a “false self” (Jørgensen 2006, p. 635) may allow BPD sufferers to navigate their social environments, but it comes at a price: inauthenticity hardly enables feelings of genuine connection. Wearing a mask in order to function socially does not precisely include the feeling of a real encounter with the other, as one is withdrawing and holding back how one really feels and who one really is.

#### BPD-Related Styles in Social Intentionality

I want to provide a last example of experiential loneliness in BPD. It concerns the way people with BPD often relate to other persons. Given the many ways in which BPD sufferers are isolated from others, they often feel strong cravings for deep connection. These cravings typically persist during times in which they are destructive and can even boycott and sabotage their own relationships: “Relationships? Blah. I will only destroy them. But at the same time I feel it is what I need” (Edwards 2015, p. 67). The destructivity that accompanies social desires makes it hard for any relationship to stabilize. But that is not the only problem. Rather, it’s the specific way social desires are often structured in people with BPD. As mentioned before, while in most people, loneliness can instigate social desires in a way that makes them open to others, in BPD, the social desires often take a form that is directed at an idealized fantasy of connection. That is, craving for deep connection, people with BPD tend to have a fixed—though vague—idea of perfect and harmonious connection with others. Thus, their social desires are more concerned with an ideal relationship than with concrete others, making it difficult for others to compete with the fantasy of the ‘perfect’ others. In other words, given the focus on an ideal of connection, real relationships are prone to disappoint and fall short, making it once again hard for BPD sufferers to feel real encounters because they implicitly measure any relationship against an unachievable ideal (Pazzagli and Rossi Monti 2000). Combined with feelings of emptiness and a lack of self-feeling, individuals with BPD develop a strong awareness of the discrepancy between their perceived relationships with concrete others and the ideal relationships they crave and strive towards. A person whose social style is oriented toward perfect relationships is likely to feel chronically frustrated. This can lead to a sense of being deprived of the social goods they desire and the notion that this is because others fail to meet the expectations of an ideal relationship.

#### BPD, Experiential Styles, and Loneliness

In the past sections, I have argued that different styles in processing affective experiences, self-experiences, and other-related experiences make it difficult for BPD sufferers to feel a connection with others. I specifically focused on the styles that clearly contribute to feelings of loneliness sooner or later. I hope that these relatively intuitive cases will help us to better understand the relationship between experiential style and experiential loneliness. But first I should clarify how I conceive of BPD as a specific existential style, or, better, a set of experiential styles. I said that impulsivity, strong affect and difficulties in regulating one’s own feelings that often remain puzzling for BPD sufferers present a set of experiential styles. Similarly, ways of experiencing oneself and others are experiential styles. These are different though often related styles that can often be found in people with BPD. It should be noted however that the combination of these styles can vary in the individual case. Some individuals may have stronger difficulties in regulating their affects, others may predominantly suffer from emptiness and lack of self-feeling. The specific phenomenology of BPD is unique to each individual, and some styles may be more significant in their experience of the world than others. When I refer to BPD-related styles, I mean that these styles are likely to be a part of a person's experience of BPD. Accordingly, the idea is that BPD comes with certain experiential styles. One might also say that BPD *is* a way of experiencing the world and thus an experiential style, which is constituted by different styles that are typical for BPD. Still, it is important to note that BPD cannot be reduced to experiential styles, as contexts, social environments, and other etiological factors need to be taken into account too. Yet, it is safe to say that individuals experiencing BPD show an overlap in how they experience self, their emotions, and other people and that certain styles therein are more typical and widespread across people living with BPD than in persons with other mental health conditions.

Now, how do these styles relate to experiential loneliness? What I suggest is that these styles, in themselves, imply a form of loneliness because these styles, in themselves, are isolating. Lacking self-feeling and being overwhelmed by non-controllable emotional feelings is by itself a disruption of interpersonal synchrony and connection. Other people, with more typical and common styles of experiencing the world, do not share the emotional experience BPD sufferers are undergoing when the mentioned experiential styles are actualized. The different levels of intensity of emotion alone will create a hiatus between people involved in a situation. Furthermore, the fantasy of a perfect relationship, by itself, disturbs the real encounter with another individual and undermines the possibility of co-creating a concrete and unique relationship.

The experiential styles thus imply or *are* a form of loneliness, *experiential* loneliness. They cut off the individual from others once they become dominant in shaping a person’s experience of the world. Experiential loneliness is not to be structurally separated from the experiential styles on which they are based. Rather, experiential loneliness consists of certain experiential styles. Experiential loneliness is a label for a group of experiential styles, each of which, on its own or in combination with others, can render a person incapable of feeling or establishing connections with others as long as the specific style persists in shaping the person's experience of the world. Accordingly, I conceive of the relationship between styles and experiential loneliness as mereological. There are numerous possible styles of experiencing the world, and some of them constitute experiential loneliness which tends toward feelings of loneliness. It should be emphasized again, however, that the mentioned BPD-related experiential styles are not the only ones constituting experiential loneliness. Experiential structures that undermine felt social encounters can take many forms. I will now finalize the paper by briefly discussing two further examples to illustrate this variety.

### Further Examples: Social Anxiety and Chronic Loneliness

There is evidence of a bi-directional correlation between social anxiety and loneliness, with studies showing that social anxiety can lead to loneliness and vice versa (Oren-Yagoda et al. 2022; Maes et al. 2019). It is easy to imagine how the fear of social interactions may lead to avoidance behaviour, resulting in sufferers being left behind and alone. However, feelings of social anxiety themselves can make it hard to feel connected with others because discomfort with others can lead to unfulfilling encounters. It’s as if social anxiety blocks relevant interpersonal emotional exchange. The fear throws people back on themselves and undermines affective synchrony and sharing with others. Rather than opening up to others by exchanging thoughts, feelings, or desires and by listening to what others communicate, socially anxious people are concerned with complying with what they take to be the right or expected way of intersubjective encounters. Trying to navigate their social environments they aim to adapt to a certain image of interpersonal relationship. One might say, in the social situation, they are overly concerned with what could be identified as the Heideggerian “they” (“*das Man*”) (Heidegger 1996, pp. 107–122), i.e., specific sets of practices that are deemed appropriate and ‘normal’ for certain contexts. As a consequence, their intentional focus is on themselves and how they might be perceived by others, which may make them self-conscious and insecure. Their attention becomes primarily self-directed, while other people tend to be only perceived as a source of cues that indicate the challenges to which the person needs to respond. Like BPD sufferers, socially anxious individuals are thus oriented towards an ideal encounter. However, their focus is not on how others seem to fail to play their part. They are concerned about their own failures in bringing the social encounter to fruition. In social anxiety disorder, social desires are intermingled with deep fears in such a way that any directedness at connection will be accompanied by discomfort and stress, making it hard for social desires to be fulfilled.

But there are also other ways in which social desires can be compromised. As a last example, I want to discuss chronic loneliness. Chronic loneliness is typically defined by pervasive and lasting feelings of loneliness (Shiovitz-Ezra and Ayalon 2009). However, as Krueger and Roberts (2021) point out, we can imagine an extreme case of chronic loneliness in which people, as a result of factual isolation, can cease to feel any desire for social connection. Krueger and Roberts (2021, p. 16) circumscribe the psychological structure of a person suffering from severe and long-lasting chronic loneliness: “She will not attend to social goods in thought and memory, nor hope to be the recipient of others’ goodwill. And she will no longer expect to be able to express herself as a social agent, nor to adopt those interpersonal roles that once meant so much to her—she will not aspire to be the nurturing friend, the shoulder to cry on, the confidante, and so forth.” In such an imaginable extreme case of chronic loneliness, the individual has lost their desire for social connection. Krueger and Roberts speak of a general “affective flattening” (2021, p. 16) and compare the experiential and emotional horizon of the chronically lonely person with conditions found in people that live with depression and other mental health issues. In such conditions as well as in chronic loneliness the way the possibility of social connection features in people’s experience is significantly transformed. It ceases to be part of their individual horizon of possibilities. In fact, connection is experienced as the impossible, something at which directing one’s desires would necessarily amount to frustration. The habituation of repeated frustration of the desire for connection transforms how the chronically lonely relates to others, making connection impossible for them in a way that renders them incapable of deriving meaning through encounters with others. The social goods and the quality of relationships are eradicated from the lonely lifeworld; they cease to be something that could carry any meaning. We are dealing here with a case of experiential loneliness resulting from pervasive and repeated feelings of loneliness. The experience of the world, oneself, and others is structurally altered in a way that prevents the lonely from enjoying connections with others. Although explicit feelings of loneliness may have dissipated, the persisting condition of experiential loneliness in chronic loneliness might give rise to other painful feelings of emptiness, meaninglessness, and existential futility. How do people in such extreme cases of chronic loneliness, as depicted by Roberts and Krueger (2021), relate to the formal object of the emotion of loneliness, i.e., how do they relate to the unavailability of social goods? I suggest we could make sense of it in the following way: The stance towards the unavailability of social goods, in these cases, is one of dampening down any affective directedness at it. The lonely have learned to suppress and annihilate all social desires; they have learned to reject the goodness of social goods. It is through this “magical” transformation, as we could say with Sartre’s theory of emotion in mind (Sartre 1948/1993, pp. 64, 86), that the unavailability of social goods becomes irrelevant, mitigating the pain of felt loneliness at the price of becoming unaffectable by others and by any meaning that is socially constituted.

## Conclusion

In this paper, I have argued in favour of a pluralist view about loneliness, suggesting that there are different kinds of loneliness that can take many forms. To yield a pluralist notion of loneliness, I discussed two amendments of the standard view of loneliness. According to the standard view, loneliness is an emotion that is experienced and intentionally directed at the discrepancy between desired and real social relationships. The first amendment consisted in specifying the intentional attitude at stake by determining it as a pro-attitude towards social goods and connection as well as by defining the formal object of loneliness-qua-emotion in terms of the lack of social goods. The second amendment concerned the widespread tendency across researchers to define loneliness as an emotion. It consisted in emphasizing that loneliness need not always be *felt* loneliness as an intentional emotion. Loneliness can also manifest as a mood, existential feeling, or vague background feeling without being intentionally and explicitly *directed at* or *about* the absence of fulfilling relationships. Proposing a pluralist view of loneliness, I highlighted three assumptions: (1) that the causes of loneliness in an individual case inscribe themselves into how loneliness is experienced; (2) that the experience of loneliness varies phenomenologically; (3) that all phenomena of loneliness share a family resemblance that is defined by the fact that all phenomena relate to the formal object of loneliness-qua-emotion in one way or another.

With this pluralist view as the conceptual background, I introduced the notion of *experiential loneliness*. Experiential loneliness is defined as the experiential styles that undermine the development of social connections and, by the same token, have a tendency to develop feelings of loneliness. However, as emphasized earlier, these styles that motivate feelings of loneliness need not lead to explicit and full-blown loneliness-qua-emotion. Rather, they may give rise to existential feelings and background feelings in which connection or the lack thereof need not become a thematic object.

To illustrate the notion of experiential loneliness, I discussed typical experiential styles found in people with borderline personality disorder. I argued that alexithymia, emotional contagion, hypersensitivity, impulsivity, and external locus of control have significant repercussions for interaffective processes to the effect that felt connection with others becomes unlikely and inhibited. Moreover, I suggested that BPD instability in a person’s sense of identity and lack of self-feeling makes it difficult to establish a connection with someone else. For, connection essentially involves that *I feel myself* connected to another person. The interaffective encounter is one that occurs between different poles of feeling. To form part of the encounter, one must be able to identify with one of the poles. But any real identification must be a felt one. Yet, with unclear affects and without self-feeling the identification fails to materialize. Accordingly, it is unlikely to feel that one's own feelings contribute to the interaffective exchange due to diffuse affects and lack of self-feeling. In addition, I argued that people with BPD tend to idealize relationships, compromising their felt social desire and directing it towards a fantasized and unattainable ideal of connection. By focusing on an image of relationship, individuals with BPD miss out on the opportunity to develop real and unique relationships that are based on shared life stories.

Lastly, discussing the case of social anxiety disorder and chronic loneliness, I argued that experiential loneliness can take different forms. Future research is needed to further examine the varieties of experiential styles across different mental conditions, such as autism spectrum disorder or bipolar disorder, which may constitute distinct types of experiential loneliness. For instance, in the past years, research has shown significant overlaps between ASD and BPD (Gordon et al. 2020; Cheney et al. 2023). Indeed, instabilities in self, affect, and social intentionality can be found in ASD too. People diagnosed with ASD may, for instance, show difficulties in identifying emotions, empathizing with others, or maintaining a stable self-feeling. Just like in the case of BPD, these styles constitute a form of experiential loneliness, which may explain the feelings of loneliness that are often described by individuals with ASD (Umagami et al. 2022).

One question for future research is whether instabilities in self, affect, and social intentionality in people with ASD are qualitatively the same as for people with BPD. My suggestion is that the notion of experiential loneliness may serve as a tool to address this important question and to distinguish the different phenomenologies involved. For instance, impulsivity and emotional dysregulation in ASD may stem from sensory overload, while in BPD, hard-to-handle feelings likely arise because a person is overly sensitive to interpersonal cues. In both cases, the affective styles have an isolating effect, but the causes are different. According to the pluralist understanding of loneliness defended in this article, the causes of loneliness inscribe themselves into the experience. Now, a possible way to make this plausible is by examining how experiential styles in different conditions translate into experiential loneliness.

I want to consider the example of affective instability to briefly illustrate this point. Both ASD and BPD may involve affective instability, but the resulting experiential loneliness may differ depending on the underlying reasons. For instance, feeling cut-off because one feels hurt by the actions of others and overwhelmed by related emotions, as is often the case in BPD, is phenomenologically distinct from feeling cut-off because one is overwhelmed by the quantity of sensory information, which is perhaps more likely in ASD. Although both conditions may exhibit experiential styles such as affective instability and dysregulation, the type of experiential loneliness may differ because of the distinct contexts and reasons for each condition. If we look solely at affective instabilities and how they manifest for others, it may be hard to track the phenomenological differences in the affective processes between BPD and ASD. However, if we look at how affective instabilities in each case, as experiential styles, translate into very specific forms of experiential loneliness, we may recognize that even the seemingly similar affective instabilities in BPD and ASD are really phenomenologically heterogeneous and that they each have the potential to give rise to quite different feelings of loneliness.

Whether or not the notion of experiential loneliness will indeed be useful in distinguishing mental conditions such as ASD and BPD and the loneliness involved is something to be determined by future research. It will certainly require taking into account all the distinct styles and their combinations that typically shape people’s experience of the world in ASD and BPD.

Finally, the concept of experiential loneliness may potentially also serve as a tool in improving our understanding of loneliness-generating psychopathologies and help to develop new ways of aiding people in navigating the social world. For, thematizing the experiential styles that make it difficult for an individual to fully participate in deeply felt and stable social bonds will also help to identify measures that could improve the situation. New therapeutic approaches could be developed that address loneliness-generating aspects of a person’s style of experiencing the world. Moreover, examining an individual’s specific styles of experiencing the world will also allow clinicians to draw conclusions with regard to the circumstances under which social connection actually might be possible for them. That is, while non-typical ways of experiencing the world may make it hard for non-neurotypicals to connect in the way neurotypicals do, changing the way how the world is structured and how neurotypicals approach persons who experience the social in alternative ways may help in creating more inclusive interpersonal environments (Krueger and Maiese 2018; Schmidt 2021b). Investigating people’s *experience* of the social world thus can allow societies to reshape the social world in a way that is more inviting to the diversity of individuals.

## Footnotes

1 Some have emphasized the need to look at how the social world is set up (Krueger & Maiese 2018). Individuals with ASD may not only be cut off due to how their minds work but also due to how their social environment is structured and offered to them.
